# Summary of Transactions of the College of Physicians of Philadelphia, from May to October, 1845, Inclusive

**Published:** 1846-01-01

**Authors:** 


*Summary of the transactions of the College of Physicians of Philadelphia, from May to October*, 1845, *inclusive.*—One of the most important of the means by which Medical Science is to be advanced, and the character of the Profession elevated, is the formation of Associations for the promotion of these objects. In nearly all the States, Medical Societies are already formed, but their chief end has been to maintain some organization as a profession—to retain some of the landmarks by which its limits are defined. Their objects, in other words, have been merely conservative, relating mostly to measures necessary to protect ourselves against encroachments upon our rights and privileges as a profession, and to preserve, as such, in some degree, that distinctive character and position upon which depend to a considerable extent our dignity and usefulness. These objects are by no means unimportant; but there are others of greater importance, and by securing the latter, the former will also be promoted. We need associations for the interchange of ideas, the reciprocal communication of the results of individual experience and observation; encouragement of scientific inquiries, and profound attainments; the collection and diffusion of progressive medical knowledge, &c. We should be glad to see a National Association formed, which should take cognizance of these objects. We believe such an association, if well-organized, and judiciously managed, would do vastly more in furtherance of the progress of science, and to raise the standard of our profession, than any legislative enactments which we can ever hope to procure, perhaps more than legislation could do if its action were placed entirely at our disposal. A National Association, such as our imagination represents, would not only develope and cultivate medical science and literature, but would give to them a nationality of character, which, as Americans, we ought in justice to ourselves to consider. We do not purpose to follow out reflections upon this subject at this time. We were led to what we have written by the caption prefixed to this article. It is the title to a periodical of about 40 pages, which appears to be published semi-annually. This is the second number which we have received. The College of Physicians of Philadelphia, as we gather from a perusal of these transactions, is an association of distinguished members of the Profession in the ancient medical metropolis, which elects associates and corresponding members in different parts of the country.—It holds monthly meetings for the presentation of communications, reports, and the discussion of subjects pertaining to medicine and surgery. It has a library, and a reading room containing all the medical periodicals of our country. The published transactions contain the papers communicated, voluntarily, or by appointment, with the remarks elicited thereupon at the meetings of the College. The plan and purposes of the institution are excellent, and might, with advantage, be imitated in other places—above all, as we have said, we should rejoice to see a National Association embracing similar objects, and we trust that if the project of a national convention succeed, this will be one of its results. We proceed to give a brief synopsis of some of the transactions relating to practical medicine:

On the subject of *Hydrarg. Sub-sulphate, or Turpeth mineral, as a remedy in some of the diseases of children*, a communication was received from Dr. Hubbard, of Hallowell, Me., Dr. H. strongly recommends this preparation as an emetic, especially in Croup. He states that he has used it extensively for the last ten years. Its advantages are these: it is reliable, prompt, efficient, and safe as an emetic, and not liable to act as a cathartic in the place of producing emesis. The objections to antimonials in young children, can be appreciated by those practitioners who have ever witnessed the alarming prostration which occasionally results from their use. Although active in its operation Dr. H. considers it entirely safe. He says, "I have never known it to be violent, nor otherwise than entirely safe in its operation, although I have given it in much larger doses than are usually directed; nor have I ever seen salivation follow its use as an emetic. So safe do I consider it, that in urgent cases I have not hesitated to put my patient under its full emetic operation two or three times within twenty-four hours; nor have I seen ill-consequences from such practice. From two to three grains may be given to a child two years old, and repeated in ten or fifteen minutes, until emesis is produced. If the first dose fail, the second usually acts as soon as it touches the stomach."

We fancy this practice is not peculiar to Dr. H., although, of course, the testimony of his experience is valuable. Some ten years ago we recollect hearing a physician of extensive practice in the centre of the State (the late Dr. Wells of Canandaigua) speak in warm terms of the use of this remedy in croup. He stated that in his neighborhood, it was almost regarded in the light of a specific in this disease. We have, also known it prescribed by a senior member of the profession in this city. Croup, however, is not a frequent disease in this locality. Considering the coldness of the climate, and the sudden alterations of temperature to which we are exposed, this is a singular fact. We cannot recal a dozen cases which have come to our knowledge in this city for the last ten years; and not a single fatal case in this period has occurred in our own practice.

*With regard to the use of Antimony in Children in pectoral affections.*—The remarks of Dr. Condie succeeding the communication of Dr. H. are valuable. It is not only liable to injure by its immediate sedative effects, but when often repeated, and long continued, as it frequently is, especially in the form of Coxe's Hive Syrup, its direct action on the lining membrane of the stomach is highly injurious. The authors of a late French work on the diseases of children (Rilliet and Barthez,) of high authority, state that acute gastritis, and even pustulation of the mucus membrane may be produced in this way.

*On the treatment of Gonorrhœa by Tartarised Antimony.*—Dr. Ashmead stated he had made a series of observations in the venereal wards of the Philadelphia Hospital, "Four patients affected with gonorrhœa were treated with tartarised antimony alone. They were placed under the use of one thirty-second of a grain, in solution, every hour—the dose being gradually increased to one-eighth of a grain at the same intervals. The disease, in every case, rapidly disappeared under this treatment—no one lasting more than a week, while in one of them, it was said by a gentleman in attendance to have terminated in forty-eight hours. In two of the cases, the gonorrhœa was complicated wilh inflamed testicle, the only application to which, was a single poultice of bread and water."

The results of these observations are unsatisfactory, from the stage of the disease at which this practice was commenced not being stated. The regimen and diet should also have been reported. In hospital practice, where patients are subject to our control, this method may succeed; and, indeed, regimen and diet alone, would, probably, often suffice; but in private practice, we much doubt if it will supersede other measures.

This number of the Trausactions contains a biographical notice of the late Dr. John Ruan, of Philadelphia, by Dr. Bond, and an annual retrospective report of some length, on *the theory and practice of medicine*, by Dr. Jackson, formerly of Northumberland. We cannot do justice to the latter without making a more extensive analysis than will comport with our space. To the readers of the American Journal of Medical Sciences, Dr. Jackson is well known as a man of thought, experience, and erudition, and a writer of unusual vivacity and terseness. His retrospective report is interesting, and we should gladly make extracts if our limits permitted.

